# Supplementary material for: Solar water splitting by photovoltaic-electrolysis with a solar-to-hydrogen efficiency over 30%
Source: Nat Commun. 2016 Oct 31;7:13237. doi: 10.1038/ncomms13237 (PMC5095559; doi:10.1038/ncomms13237)
Supplement: Supplementary Information — Supplementary Figures 1-3, Supplementary Table 1, Supplementary Notes 1-4 and Supplementary References. [file ncomms13237-s1.pdf]

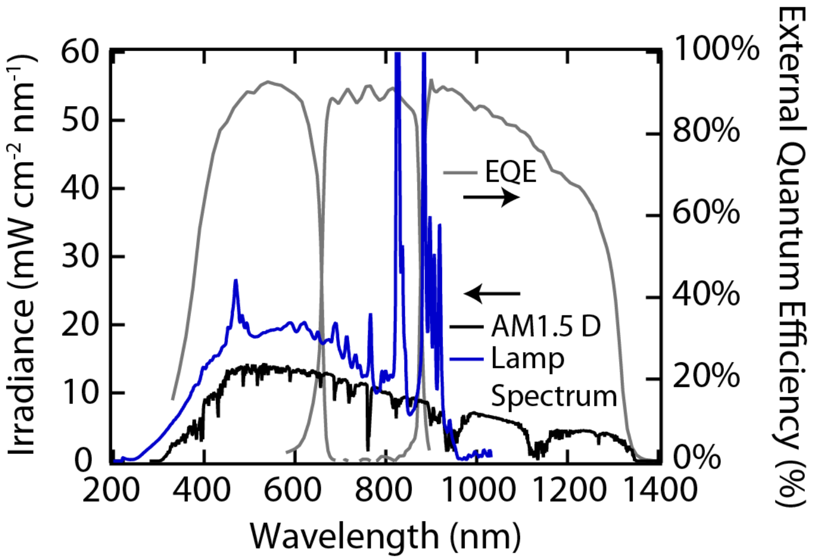

Supplementary Figure 1. Reference spectrum AM 1.5D, spectrum for multi-sun Newport xenon arc lamp, and external quantum efficiency. The lamp spectrum is the output of the Newport Model 66921 1000 W xenon arc lamp used as a multi-sun solar simulator in this study. This spectrum is compared to the AM 1.5D reference spectrum. The external quantum efficiency curve was measured for 3J photovoltaic used in these experiments as described in the methods section.

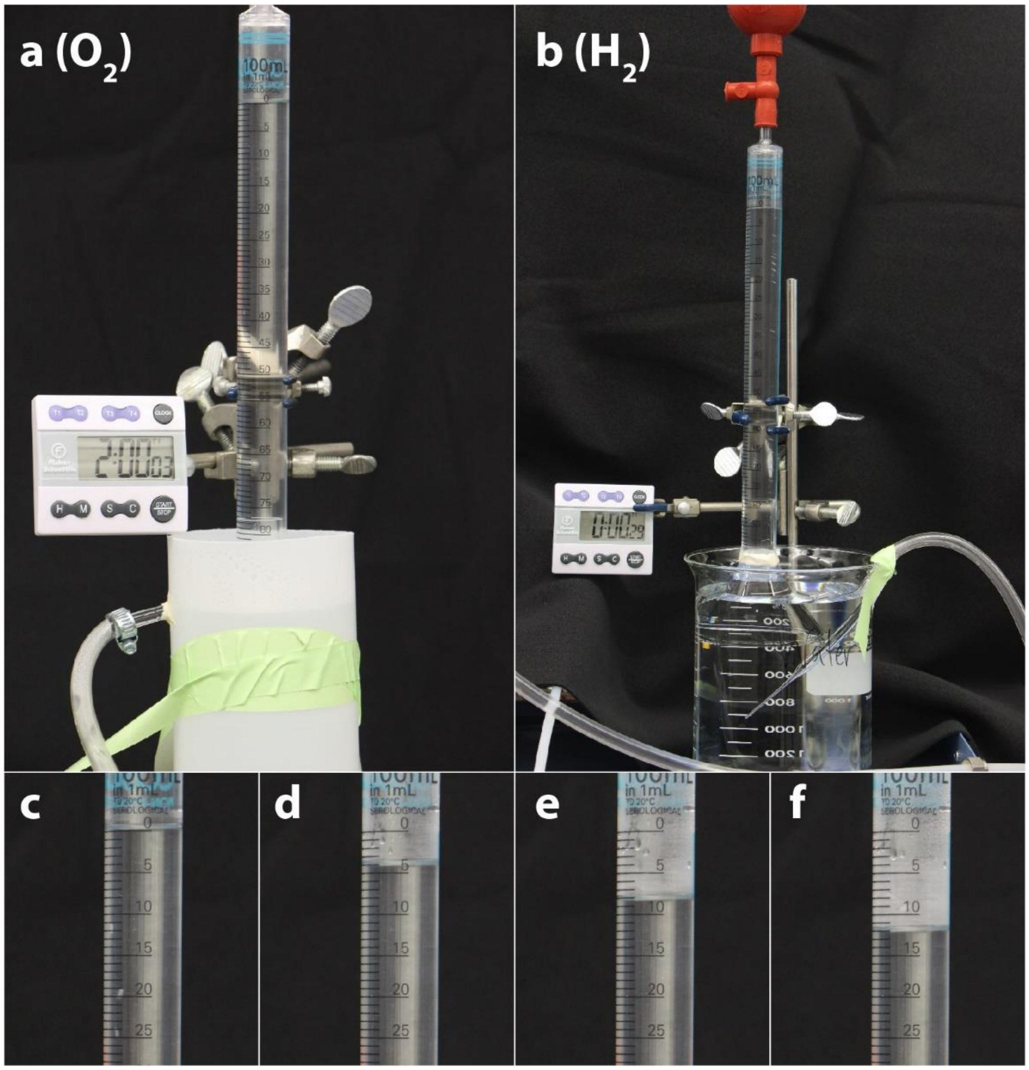

Supplementary Figure 2. Photographs of Faradaic efficiency measurement apparatus.
a. The O_2 product and unreacted water were flowed into a plastic beaker, where the gas bubbles were collected in a 100 mL pipette for volume measurement and the extra water was removed to prevent the apparatus from overflowing. **b.** The H_2 product was bubbled into a separate 100 mL pipette for volume measurement. **c-f.** Example photographs showing how the volume was measured to create plots such as those shown in Supplementary Figure 3.

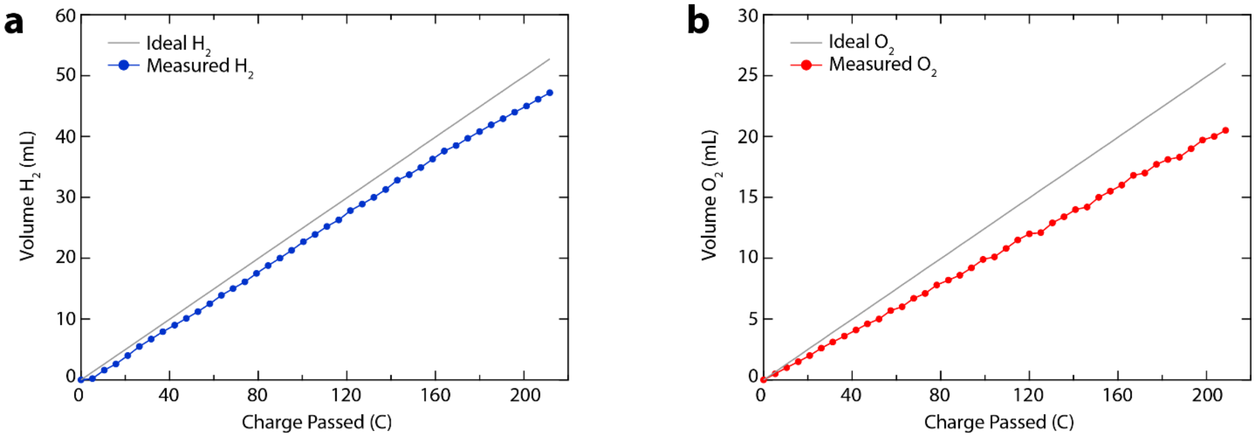

Supplementary Figure 3. Representative Faradaic efficiency data. a. The H_2 Faradaic efficiency measured during this 20 min trial was 90%, and the average H_2 Faradaic efficiency measured over the 48 hour experiment was 92%. **b.** The O_2 Faradaic efficiency measured during this 20 minute trial was 79%, and the average O_2 Faradaic efficiency measured over the 48 hour experiment was 78%. The H_2 and O_2 volumes produced by the photovoltaic-electrolyzer were measured as described above. The ideal H_2 and O_2 volumes were calculated by assuming 100% Faradaic efficiency for these products.

**Photocurrent (mA cm^{-2})**	J_{top}	J_{mid}	J_{bottom}	J_{SC}
AM 1.5D	14.39	13.66	14.92	13.89
Multi-sun Solar Simulator	14.83	21.29	11.75	11.86

Supplementary Table 1. Comparison of modeled and measured photocurrents. Photocurrent of each subcell when modeled with AM 1.5D spectrum and measured under the multi-sun solar simulator. The currents are normalized such that they represent the value as the input light has a spectrum intensity of 100 mW cm^{-2}.

Supplementary Note 1:

Calculation of simulated solar concentrations. The spectrum used in standard reporting conditions (SRC) for concentrator solar cells is defined as the AM 1.5D spectrum scaled by the ratio of short-circuit current (J_{SC}) at high irradiance to the 1-sun value.[1] It is therefore a common practice to determine the simulated solar concentration from the J_{SC} ratio.[1,2] In this work, since the 1-sun simulator was calibrated to AM 1.5D spectrum, the simulated solar concentration, or number of suns, was determined by taking the ratio between the multi-sun J_{SC} and the 1-sun J_{SC}. As shown in Figure 2, a J_{SC} of 13.9 mA cm^{-2} was measured under the one-sun simulator and 583.6 mA cm^{-2} was measured under the multi-sun simulator for the 48-hour PV-electrolysis measurement. Therefore, it was calculated that the cell was exposed to approximately 42 suns of incident light during demonstrated operation.

Supplementary Note 2:

Faradaic efficiency measurements, calculations, and discussion. The outputs of the anode and cathode from the second electrolyzer were collected so that the H_2 and O_2 products could be quantified using a volume displacement Faradaic efficiency measurement apparatus shown in Supplementary Figure 2. The product streams were fed separately into 100 mL pipettes filled with water and fitted with funnels at the base to catch all the bubbles. The H_2 produced at the electrolyzer cathodes was collected as a gas and the O_2 produced at the anodes was collected as gas bubbles carried along with the unreacted water. Each data point consisted of a 20 minute accumulation of H_2 and O_2 products, which enabled the calculation of the average Faradaic efficiency of H_2 and O_2 production over this duration. For some measurements, the apparatus was digitally photographed once every 30 s for 20 min. Supplementary Figure 3 shows a representative set of the resulting data. Panels (a) and (b) show the amount of H_2 and O_2 measured, respectively, along with the ideal gas volumes calculated assuming 100% Faradaic efficiency for these products. Volume displacement measurements were taken 13 times over the course of 48 hours of continuous electrolysis. The average Faradaic efficiency for H_2 was 92% and for O_2 was 78%.

There are several potential reasons why the measured Faradaic efficiency values could be lower than the ideal 100%. The most likely explanation is that some of the H_2 and O_2 gases dissolved in the water and potentially outgassed to the air. It is reasonable to expect that this process could occur to a greater extent for the O_2 compared to the H_2 because the O_2 flowed out of the electrolyzer in a two-phase mixture with water, giving it additional time and water volume in which to dissolve. It is also possible that the electrolyzer or other components of the experimental apparatus were not completely gas tight and some of the product leaked to the atmosphere. A third possibility is that the electrolyzer was actually not entirely selective for the production of H_2 and O_2, and some of the charge passed through the cell went towards other electrochemical processes. While we are not able to definitively rule out this possibility, we believe it is unlikely for several reasons:

1. The total current through the device and the Faradaic efficiency values did not change significantly through the 48 hr electrolysis. If a substantial fraction of the current through the device were going to another process such as corrosion of the electrolyzer components, we would expect to see a more substantial impact on the device performance.

2. The amount of charge passed during the experiments was large enough that competing electrochemical processes could not be sustained for the entire 48 hr. For example, consider the amount of charge that would be required to oxidize all the metal at the anode of the device. (Similar arguments could be applied to the reduction of cathode components, but it is not clear what reactions aside from the HER could occur.) The mass of Ir nanoparticles was 0.0125 g and the Ti mesh was 0.045 g. Assuming 4 electron oxidations for both metals:

$$0.0125\ g\ Ir * \frac{1\ mol\ Ir}{192.22\ g\ Ir} * \frac{4\ mol\ e^-}{1\ mol\ Ir} * \frac{96485\ C}{1\ mol\ e^-} = 25.1\ C \quad (2)$$

$$0.045\ g\ Ti * \frac{1\ mol\ Ti}{47.87\ g\ Ti} * \frac{4\ mol\ e^-}{1\ mol\ Ti} * \frac{96485\ C}{1\ mol\ e^-} = 363\ C \quad (3)$$

In comparison, the total charge passed during the 48 hr experiment was 29,290 C. Therefore, Ti and Ir oxidation could account for only 1.3% of the total charge passed, and cannot explain the measured H_2 and O_2 Faradaic efficiency values.

In light of these considerations, these Faradaic efficiency measurements provide strong evidence that the PEM electrolyzers used in this study were highly selective for the production of the desired H_2 and O_2 products.

Supplementary Note 3:

Photovoltaic-electrolyzer solar to hydrogen efficiency calculation. Power output (P_{out}) was calculated by multiplying the measured current through the two electrolyzers and solar cell in series by the thermodynamic potential required to split water at 80°C (1.18 V) and including a factor of two to account for H_2 production from both electrolyzers. The average electrolysis current for the first 20 minutes of operation was 175.6 mA so the (P_{out}) is 414.3 mW. The power input (P_{in}) was determined by the concentrated light input which was calculated to be approximately 42 suns as described previously. Multiplying this concentration by the 1-sun illumination intensity ($100\ mW\ cm^{-2}$) and the solar cell area ($0.316\ cm^2$) results in a P_{in} of $1328\ mW$. Supplementary Equation 1 shows this STH calculation for first 20 minutes of operation:

$$STH = \frac{P_{out}}{P_{in}} = \frac{2\times1.18V\times175.6mA}{42\times100mW\ cm^{-2}\times0.316cm^2} = \frac{414.3mW}{1328mW} = 31.2\% \quad (1)$$

Supplementary Note 4:

Calculation of effects of spectral mismatch. When measuring the performance of multijunction solar cells, it is crucial to determine the measurement error due to spectral mismatch.[3,4] We measured the spectrum of our multi-sun solar simulator, as shown in Supplementary Figure 1. It was observed that the bottom subcell of the solar cell generates less photocurrent than the two upper subcells when illuminated with this light source. Supplementary Table 1 shows the photocurrent of each subcell of the cell under AM 1.5D and the multi-sun lamp spectra. It can be seen that the top and middle subcells are overdriven under the multi-sun solar simulator and therefore generate excessive voltage.[3,4] To precisely determine the voltage inflation, we used the optoelectronic model described by Geisz, et al.[5] and isotype cell parameters measured at Solar Junction to predict the voltage that would be generated by this solar cell under multi-sun

illumination perfectly matching the AM 1.5D spectrum. The model shows the cell should have a V_{OC} of 3.19 V under 42 suns of AM 1.5D illumination, while the cell was measured to have a V_{OC} of 3.21 V under the multi-sun simulator with the same solar concentration. Therefore, it was estimated that there was ~20 mV of voltage inflation due to the spectral mismatch, which is too small to cause significant error to the PV-electrolysis measurement because the operating voltage of the PV driving the electrolyzers was lower than the maximum-power-point voltage of the solar cell. The slope of the solar cell I-V curve at the PV-electrolysis operating point was 0.047 mA mV^{-1} at the beginning of operation and 0.181 mA mV^{-1} at the end of operation. Using these slopes, we estimate that voltage inflation could have caused an operating current inflation of at most 0.94 mA at the beginning of operation and 3.62 mA at the end of operation. Therefore, we estimated the STH inflation due to the spectrum mismatch was at most 0.17% (abs.) at the beginning of operation and 0.73% (abs.) at the end of operation.

Supplementary References

1 Osterwald, C., Wanlass, M., Moriarty, T., Steiner, M. & Emergy, K. in *IEEE 40th Photovoltaic Specialist Conference (PVSC)* 2616-2619 (Denver, CO, 2014).

2 Osterwald, C. *Translation of Device Performance Measurements to Reference Conditions*. Vol. 18 269-279 (1986).

3 Meusel, M., Adelhelm, R., Dimroth, F., Bett, A. W. & Warta, W. Spectral mismatch correction and spectrometric characterization of monolithic III–V multi-junction solar cells. *Progress in Photovoltaics: Research and Applications* **10**, 243-255, doi:10.1002/pip.407 (2002).

4 Siefer, G. *et al.* in *Photovoltaic Specialists Conference, 2002. Conference Record of the Twenty-Ninth IEEE.* 836-839.

5 Geisz, J. F. *et al.* Generalized Optoelectronic Model of Series-Connected Multijunction Solar Cells. *IEEE Journal of Photovoltaics* **5**, 1827-1839, doi:10.1109/jphotov.2015.2478072 (2015).
